# Burnout and resilience among Canadian palliative care physicians

**DOI:** 10.1186/s12904-020-00677-z

**Published:** 2020-11-06

**Authors:** Cindy Wang, Pamela Grassau, Peter G. Lawlor, Colleen Webber, Shirley H. Bush, Bruno Gagnon, Monisha Kabir, Edward G. Spilg

**Affiliations:** 1 Department of Medicine, Division of Palliative Care, University of Ottawa, 43 Bruyère St., Ottawa, ON K1N 5C8 Canada; 2 School of Social Work, Carleton University, 1125 Colonel By Dr., Ottawa, ON K1S 5B6 Canada; 3 Department of Medicine, Division of Palliative Care, Bruyère Research Institute, and Ottawa Hospital Research Institute, University of Ottawa, 43 Bruyère St., Ottawa, ON K1N 5C8 Canada; 4 Ottawa Hospital Research Institute and Bruyère Research Institute, 43 Bruyère St., Ottawa, ON K1N 5C8 Canada; 5 Palliative Care, Department of Family Medicine and Emergency Medicine, Cancer Research Centre, Laval University, 6, rue McMahon 1899-6, Québec, Québec G1R 2J6 Canada; 6 Bruyère Research Institute, 43 Bruyère St., Ottawa, ON K1N 5C8 Canada; 7 Department of Medicine, Division of Geriatric Medicine, University of Ottawa and Ottawa Hospital Research Institute, 1053 Carling Avenue, Ottawa, ON K1Y 4E9 Canada

**Keywords:** Burnout, Resilience, Palliative care physicians

## Abstract

**Background:**

Physicians experience high rates of burnout, which may negatively impact patient care. Palliative care is an emotionally demanding specialty with high burnout rates reported in previous studies from other countries. We aimed to estimate the prevalence of burnout and degree of resilience among Canadian palliative care physicians and examine their associations with demographic and workplace factors in a national survey.

**Methods:**

Physician members of the Canadian Society of Palliative Care Physicians and Société Québécoise des Médecins de Soins Palliatifs were invited to participate in an electronic survey about their demographic and practice arrangements and complete the Maslach Burnout Inventory for Medical Professionals (MBI-HSS (MP)), and Connor-Davidson Resilience Scale (CD-RISC). The association of categorical demographic and practice variables was examined in relation to burnout status, as defined by MBI-HSS (MP) score. In addition to bivariable analyses, a multivariable logistic regression analysis, reporting odds ratios (OR), was conducted. Mean CD-RISC score differences were examined in multivariable linear regression analysis.

**Results:**

One hundred sixty five members (29%) completed the survey. On the MBI-HSS (MP), 36.4% of respondents reported high emotional exhaustion (EE), 15.1% reported high depersonalization (DP), and 7.9% reported low personal accomplishment (PA). Overall, 38.2% of respondents reported a high degree of burnout, based on having high EE or high DP. Median CD-RISC resilience score was 74, which falls in the 25th percentile of normative population. Age over 60 (OR = 0.05; CI, 0.01–0.38), compared to age ≤ 40, was independently associated with lower burnout. Mean CD-RISC resilience scores were lower in association with the presence of high burnout than when burnout was low (67.5 ± 11.8 vs 77.4 ± 11.2, respectively, *p* < 0.0001). Increased mean CD-RISC score differences (higher resilience) of 7.77 (95% CI, 1.97–13.57), 5.54 (CI, 0.81–10.28), and 8.26 (CI, 1.96–14.57) occurred in association with age > 60 as compared to ≤40, a predominantly palliative care focussed practice, and > 60 h worked per week as compared to ≤40 h worked, respectively.

**Conclusions:**

One in three Canadian palliative care physicians demonstrate a high degree of burnout. Burnout prevention may benefit from increasing resilience skills on an individual level while also implementing systematic workplace interventions across organizational levels.

**Supplementary Information:**

The online version contains supplementary material available at 10.1186/s12904-020-00677-z.

## Background

Burnout, the outcome of extensive job-related stress, is characterized by depletion of emotional resources (emotional exhaustion [EE]), feelings of cynicism (depersonalization [DP]), and a sense of diminished personal accomplishment and self-achievement (personal accomplishment [PA]) [1–3]. Burnout in health care professionals has been associated with poor mental and physical health [4–7], lower quality of patient care [8–10], more medical errors [11–15], and lower empathy [13, 14, 16–18]. Finally, burnout has significant impact on physician absenteeism and retention [19–22].

In a 2017 survey of US physicians, 43.9% reported burnout and the prevalence of burnout symptoms across specialties ranged from 29.6% (preventative medicine/occupational medicine) to 54.9% (emergency medicine) [23]. With respect to palliative care physicians in the United States, burnout prevalence rates have been reported as ranging from 33 to 38% [24, 25] in addition to a more recent clarification on an earlier reported study reflecting 38.7% [26, 27]. Similar work on burnout in palliative care physicians in other countries have been reported as 41.9% in Singapore [28] and 24% in Australia [29]. In a French study of palliative care physicians, the overall burnout rate was not measured but the rates of high emotional exhaustion and high depersonalization were considerably lower at 9 and 4% respectively [30]. To date, there is no published comparative data for Canadian palliative care physicians.

Resilience, a multidimensional construct that reflects the personal qualities that enable an individual to adapt and grow in the face of adversity [31], is a factor that can mitigate and potentially even protect people from burnout [32–34], Resilience building interventions have been shown to be helpful as part of a skill-building approach to stress reduction in palliative care clinicians [34, 35]. Studies examining physician burnout have elucidated some individual and work-related protective and risk factors for burnout. Protective factors include being older [23, 29], having worked more years [29, 36], and being married [23, 28]. Risk factors include being female [23, 28, 36, 37], increased hours worked per week [23, 28, 37, 38], and working in certain high acuity specialties such as emergency medicine [23]. Some palliative care specific protective factors include working exclusively in palliative care [29], being spiritual [28], and working with more colleagues [38]. There are mixed results regarding whether working in community or hospital-based palliative care is associated with higher risk of burnout [28–30].

Collectively, exploring Canadian palliative care physician experiences of burnout and resilience provides an important body of literature, as there are clear gaps remaining [32, 33, 35, 39]. In order to address this gap, we conducted a national cross-sectional survey to better understand the prevalence of burnout and degree of resilience experienced by Canadian palliative care physicians and to examine the relationship between burnout, resilience, demographic and job factors.

## Methods

We conducted an electronic survey of demographics, burnout and resilience among palliative care physicians in Canada. Participation was voluntary and no remuneration was provided. The study was approved by the Bruyère Research Ethics Board and the Ottawa Health Sciences Network Research Ethics Board. Informed written consent was obtained from each participant. Survey planning and administration was reported as per the “Checklist for Reporting Results of Internet E-Surveys” (CHERRIES) [40].

### Participants

Physicians and resident physicians who were active members of Canadian Society of Palliative Care Physicians (CSPCP) or Société Québécoise des Médecins de Soins Palliatifs (SQMDSP) as of March 18, 2019 were invited via email to participate. Members who were non-physicians, retired physicians, and physicians working in countries outside of Canada, and physicians and resident physicians from CSPCP who had not authorized research participation requests were excluded from the invitation email. Details of the survey’s sampling frame are presented in a supplementary figure (Additional file 1), which presents the derivation of the total of 569 physicians from the respective societies’ membership lists to whom invitation emails were sent: 479 members in the CSPCP and 90 members in the SQMDSP.

### Survey procedures

Over the course of 5 weeks (between March 18th, 2019 and April 23rd, 2019), administrators of CSPCP and SQMDSP distributed an initial invitation email (followed by two reminders), with embedded and secure SurveyMonkey® electronic links to both English and French versions of the survey.

### Survey measures

The survey, inclusive of questionnaires, consisted of 57 questions in total, and of these a set of 10 questions concerned participant demographics: clinical practice, clinical settings, training and remuneration arrangements. These demographic questions were developed by the research team in English and translated to French by a certified translator. The last question of the survey, ‘Thank you for your participation. Leave a comment if you wish”, provided participants with the option of inserting informal free text comment.

Burnout was measured using the Maslach Burnout Inventory-Human Services Survey for Medical Professionals [MBI-HSS (MP)], a validated 22-item questionnaire considered to be the gold standard tool for measuring professional burnout [3]. The questionnaire has 3 subscales to evaluate each domain of burnout, including emotional exhaustion (EE; 9 items), depersonalization (DP; 5 items), and personal accomplishment (PA; 8 items). Each item is rated using a seven-point Likert scale grading the extent each item is experienced by the respondent, ranging from *never* (0) to *everyday* (6). Additive scores from items under each subscale represent the total score for that subscale and are used in determining the level of risk for burnout. Based on normative data in medical professionals, EE was scored as high for total score ≥ 27; DP scored as high for total score ≥ 10; PA scored as low for a total score ≤ 33 [2]. High burnout was defined as having a high score on EE (≥27) and/or DP (≥10) subscale, in line with other studies measuring burnout in medical professionals [28, 29, 38, 41]. Resilience was measured using the 25-item Connor-Davidson Resilience Scale (CD-RISC) [31], a widely accepted measure of resilience [42]. Respondents rate their level of agreement with each scale item on a five-point Likert scale ranging from *not true at all* (0) to *true nearly all the time* (4). Possible scores range from 0 to 100, with higher total scores reflecting greater resilience. English and French-Canadian versions of the MBI-HSS (MP) and CD-RISC tools were used.

### Statistical analysis

Descriptive statistics were used to describe and examine the relationship between demographics, practice characteristics, and burnout and resilience measurements of palliative care physicians. A t-test was performed to examine differences in mean CD-RISC scores between physicians with high or low burnout. A *p*-value less than 0.05 was considered statistically significant. Bivariable chi-square tests and multivariable logistic and linear regression models were used to evaluate the associations between demographic and practice variables and the presence of burnout and resilience levels. The outcome of the multivariable logistic regression analysis was reported in terms of odds ratios (OR) and their respective 95% confidence intervals (CI). Variables chosen for primary data collection and ultimately considered for inclusion in the multivariable regression models were primarily based on literature data and those hypothesized by the research team to have the greatest effect on burnout and resilience. This selection process was further guided by the results of the bivariable analyses and the need to avoid multicollinearity. All quantitative data were analyzed using SAS v.9.3.

All open text comments were carefully reviewed and then imported into NVivo 12, a qualitative data analysis software program [43]. Drawing on an inductive approach, wherein each participants’ comments were reviewed as a specific unit of text [44], two authors, one a qualitatively trained researcher, coded the open-text into initial codes, capturing the content, meaning and context of each participant response [45, 46]. after moving between and across initial codes and exploring patterns and responses across participants, a more situated, and nuanced thematic coding process was developed [44–47]. Moving between and across initial codes, and focusing on the overall study’s research question in exploring the relationship between burnout, resilience, demographic and job factors, we began to explore patterns across the initial codes, thereby developing thematic codes which offered further insights, understanding and context into the quantitative data collected. In dialogue with the larger research team, our open-text thematic analysis was then situated in conversation with the emerging conceptual understanding of how physicians across disciplines experience burnout, resilience and wellness [48–52].

## Results

**Fig. 1:**
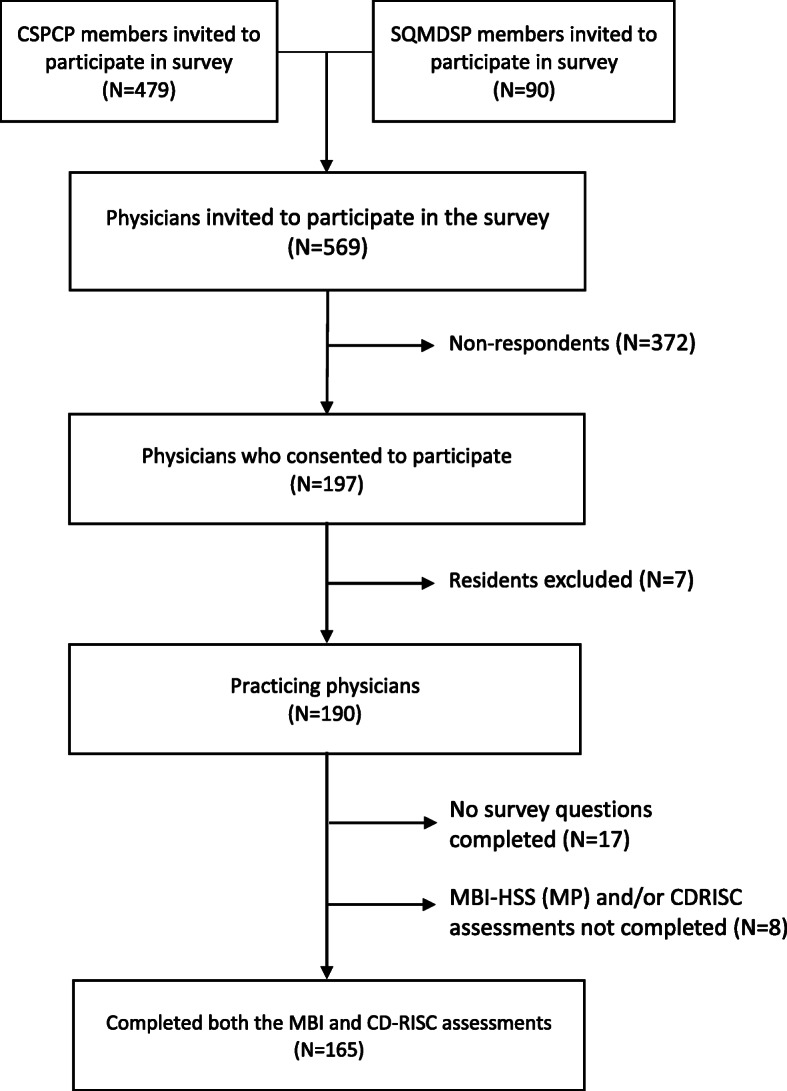
Flowchart of survey participation. **CSPCP:** Canadian Society of Palliative Care Physicians; **SQMDSP:** Société Québécoise des Médecins de Soins Palliatif; **MBI-HSS (MP):** Maslach Burnout Inventory Human Services Survey for Medical Professionals**; CD-RISC:** Connor-Davidson Resilience Scale

Of the 569 physicians who were invited to participate, survey responses were received from 197 members (Fig. 1). Resident physicians only accounted for 7 responses and so were excluded from the study. Of the remaining 190 consenting respondents, 17 did not complete any survey questions, while 173 respondents completed ≥1 survey question. Of those, 165 completed both the Maslach Burnout Inventory-Human Services Survey for Medical Professionals MBI-HSS (MP) and the Connor-Davidson Resilience Scale (CD-RISC) assessments, providing a response rate of 29% (165/569). All analyses were completed on the 165 respondents who completed both assessments.

### Respondent characteristics

Demographic and job characteristics of respondents are shown in Table 1. Almost half (49%) of the respondents were aged > 50 years and over two thirds (70%) of respondents were female. A majority of respondents (53%) worked 41–60 h per week. Most respondents worked predominantly (> 50% of clinical time) in palliative care (79%), and in a variety of settings. Approximately half of the respondents (51%) spent ≥20% of work time on non-clinical activities. Most respondents trained in family medicine/ general practice (68%). Just under half of the respondents (47%) had confirmed additional formal training in palliative care. The most frequent remuneration arrangement was a clinical or mixed clinical-academic salaried position in 44% of respondents.

**Table 1:** Respondent characteristics

Characteristics	*N* = 165 (%)
**Age (years)**	
≤ 40	48 (29.1)
41 to 50	37 (22.4)
51 to 60	51 (30.9)
> 60	29 (17.6)
**Gender**	
Male	49 (29.7)
Female	116 (70.3)
**Preferred language for survey completion**	
English	135 (78.8)
French	35 (21.2)
**Years in practice**	
≤ 10	53 (32.1)
11 to 20	38 (23.0)
21 to 30	41 (24.8)
> 30	33 (20.0)
**Hours per week**	
≤ 40	54 (32.7)
41 to 60	88 (53.3)
> 60	23 (13.9)
**Care setting**	
Palliative Care Unit	66 (40.0)
Hospice	62 (37.0)
Hospital-based consult team	101 (61.2)
Community	89 (53.9)
Outpatient clinic	20 (12.1)
**Time in clinical practice is predominantly (> 50%) in palliative care**	
Yes	131 (79.4)
No	34 (20.6)
**Spends ≥ 20% of work time on non-clinical activities**	
Yes	84 (50.9)
No	81 (40.1)
**Length of time that clinical care has focused on palliative care**	
≤ 5 years	41 (24.8)
6 to 10 years	43 (26.1)
11 to 20 years	40 (24.2)
21 to 30 years	18 (10.9)
> 30 years	7 (4.2)
Not reported	16 (9.7)
**First residency**	
General practitioner/Family Medicine	114 (69.1)
Internal Medicine	20 (12.1)
Anaesthesia	4 (2.4)
Emergency Medicine	4 (2.4)
Pediatrics	6 (3.6)
Urology	1 (0.6)
Not reported	16 (9.7)
**Has additional formal training in palliative medicine**	
Yes	77 (46.7)
No	72 (43.6)
Not reported	16 (9.7)
**Remuneration**	
Clinical and/or clinical-academic salary	73 (44.2)
Fee for Service	35 (21.2)
Combination of above	41 (24.8)
Not reported	16 (9.7)

### Burnout

The Maslach Burnout Inventory-Human Services Survey for Medical Professionals MBI-HSS (MP) includes 3 subscales, emotional exhaustion (EE), depersonalization (DP), and personal accomplishment (PA).

Table 2 shows respondents who scored low, moderate or high on EE, DP and PA based on tertiles derived from normative data. Overall, 36.4% of respondents had high EE, 15.1% had high DP, and 7.9% had low PA. We found that 38.2% of respondents had one manifestation of high burnout based on having high EE and/or high DP.

**Table 2:** Maslach Burnout Inventory Human Services Survey for Medical Professionals [MBI-HSS (MP)] and Connor-Davidson Resilience Scale [CD-RISC] scores

MBI-HSS (MP) Scores	
**Emotional Exhaustion (EE)**	
≤ 18	67 (40.6)
19 to 26	38 (23.0)
≥ 27	60 (36.4)
**Depersonalization (DP)**	
≤ 5	106 (64.2)
6 to 9	34 (20.6)
≥ 10	25 (15.1)
**Personal Accomplishment (PA)**	
≥ 40	119 (72.1)
39–34	33 (20.0)
≤ 33	13 (7.9)
**Burnout Criteria Met (EE ≥ 27 and/or DP ≥ 10)**	
Yes	63 (38.2)
No	102 (61.8)
**CD-RISC Scores**	
**Total CD-RISC**	
Mean (SD)	73.6 (12.4)
Median	74
Minimum	44
25th percentile	64
75th percentile	83
Maximum	98

### Resilience

The Connor-Davidson Resilience Scale (CD-RISC).

Table 2 shows CD-RISC scores stratified into quartiles. Median CD-RISC score of respondents was 74, which was marginally above the 25th percentile of a US normative population [53].

### Associations between burnout, resilience, and demographic and job factors

Table 3 shows bivariable associations between demographic and job variables listed and manifestation of burnout. Older physicians (> 60 years of age) were at much lower risk for burnout as compared to younger physicians (*p* = 0.0003). Similarly, there was a trend toward decreased burnout in increased years worked (> 30 years) and increased length of time spent in palliative care (> 20 years) with no statistical significance. There was a trend toward decreased burnout with working ≤40 h per week. Burnout was not significantly different for those whose clinical practice was predominantly in palliative care, nor for those who spend ≥20% of work time on non-clinical activities. Prevalence of burnout was higher for those on salary as compared to fee for service (FFS)/combination of salary and FFS (*p* = 0.04). There was no gender difference in burnout rate.

**Table 3:** Burnout status compared in relation to demographic and workplace variables (row %)

	Burnout criteria met [***N*** = 63]	Burnout criteria not met [***N*** = 102]	***P*** value
**Age**			
≤ 40	20 (41.7)	28 (58.3)	0.0003
41 to 50	18 (48.6)	19 (51.3)	
51 to 60	24 (47.1)	27 (52.9)	
> 60	1 (3.4)	28 (96.5)	
**Gender**			
Male	17 (34.7)	32 (65.3)	0.55
Female	46 (39.7)	70 (60.3)	
**Years in practice**			
≤ 10	22 (41.5)	31 (58.5)	0.16
11 to 20	17 (44.7)	21 (55.3)	
21 to 30	17 (41.5)	24 (58.5)	
> 30	7 (21.2)	26 (78.8)	
**Hours worked per week**			
≤ 40	15 (27.8)	39 (72.2)	0.14
41 to 60	39 (44.3)	49 (55.7)	
> 60	9 (39.1)	14 (60.9)	
**Time in clinical practice is predominantly (> 50%) in palliative care**			
Yes	48 (36.6)	83 (63.4)	0.42
No	15 (44.1)	19 (55.9)	
**Spends ≥ 20% of work time on non-clinical activities**			
Yes	33 (39.3)	51 (60.7)	0.76
No	30 (37.0)	51 (63.0)	
**Length of time with focus on palliative care**			
≤ 5 years	19 (46.3)	22 (53.7)	0.40
6 to 10 years	17 (39.5)	26 (60.5)	
11 to 20 years	17 (42.5)	23 (57.5)	
21 to 30 years	5 (27.8)	13 (72.2)	
> 30 years	2 (28.6)	5 (71.4)	
Not reported	3 (18.7)	13 (81.2)	
**Remuneration**			
Clinical and/or clinical-academic salary)	35 (48.0)	38 (52.0)	0.04
Fee for Service/combination	25 (32.9)	51 (67.1)	
Not reported	3 (18.7)	13 (81.3)	

Table 4 shows bivariable associations between demographic and job variables listed and mean CD-RISC score. Older physicians (> 60 years of age) reported significantly higher resilience than younger physicians (*p* = 0.03). There was no gender difference in reported resilience. While not statistically significant, there was a trend toward increased level of resilience with years in practice, increased hours worked per week and having a clinical practice predominantly focussed on palliative care.

**Table 4:** Comparison of Connor-Davidson Resilience Scale [CD-RISC] scores in association with demographic and workplace variables

	CD-RISC score Mean (SD)	***P*** value
**Age**		
≤ 40	73.6 (11.7)	0.03
41 to 50	71.6 (9.7)	
51 to 60	71.8 (14.3)	
> 60	79.6 (11.5)	
**Gender**		
Male	75.2 (13.1)	0.29
Female	73.0 (12.1)	
**Years in practice**		
≤ 10	73.8 (11.2)	0.13
11 to 20	70.2 (10.9)	
21 to 30	73.6 (13.6)	
> 30	77.3 (13.7)	
**Hours per week**		
≤ 40	72.7 (11.3)	0.15
41 to 60	73.0 (12.6)	
> 60	78.3 (13.7)	
**Time in clinical practice is predominantly (> 50%) in palliative care**		
Yes	74.5 (76.7)	0.07
No	70.2 (74.4)	
**Spends ≥ 20% of work time on non-clinical activities**		
Yes	73.1 (12.4)	0.60
No	74.1 (13.5)	
**Length of time that clinical care has focused on palliative care**		
≤ 5 years	72.3 (11.6)	0.58
6 to 10 years	75.2 (11.5)	
11 to 20 years	71.6 (13.2)	
21 to 30 years	74.0 (15.6)	
> 30 years	79.3 (15.6)	
Not reported	75.0 (9.4)	
**Remuneration**		
Clinical and/or clinical-academic salary	74.4 (11.8)	0.63
Fee for Service/combination	72.6 (13.6)	
Not reported	75.0 (9.4)	

The multivariable logistic regression analysis examined the level of independent association of demographic and workplace variables with burnout (Table 5). Of the included variables, only age > 60, (OR = 0.05; 95% CI, 0.01–0.38), compared to age ≤ 40, had an independent association with lower burnout. Multivariable linear regression was conducted to examine differences in mean CD-RISC resilience scores in association with the same demographic and workplace categorical variables (Table 6). Of the included variables, there were increased CD-RISC scores of 7.77 (95% CI, 1.97–13.57), 5.54 (CI, 0.81–10.28), and 8.26 (CI, 1.96–14.57) in association with age > 60, a predominantly palliative care clinical practice (> 50% of clinical time), > 60 h worked per week as compared to ≤40 h worked, respectively. In an alternate model that included years in practice instead of age, there was a trend [Mean CD-RISC score difference of 4.59 (CI, − 0.96-10.14)] toward an increase in resilience level with > 30 years in practice as compared to < 10 years (Additional file 2).

**Table 5:** Multivariable logistic regression, estimating the odds ratios (OR) for burnout in relation to demographic and workplace variables (*n* = 165)

	OR for burnout	95% CI
**Gender**		
Male	Ref	–
Female	0.87	0.38 to 1.95
**Age**		
≤ 40	Ref	–
41–50	1.28	0.52 to 3.11
51–60	1.15	0.51 to 2.58
> 60	0.05	0.01 to 0.38
**Time in clinical practice is predominantly (> 50%) in palliative care**		
No	Ref	–
Yes	0.61	0.25 to 1.47
**Hours worked per week**		
≤ 40	Ref	
41 to 60	1.44	0.64 to 3.22
> 60	1.17	0.37 to 3.73
**Spends ≥ 20% of work time on non-clinical activities**		
No	Ref	–
Yes	1.11	0.54 to 2.28

**Table 6:** Multivariable linear regression, estimating mean Connor-Davidson Resilience Scale [CD-RISC] score differences in relation to demographic and workplace variables (n = 165)

	Mean CD-RISC score difference	95% CI
**Gender**		
Male	Ref	–
Female	0.50	−3.82 to 4.81
**Age**		
≤ 40	Ref	–
41–50	−1.28	−6.53 to 3.97
51–60	−1.78	−6.56 to 2.99
> 60	7.77	1.97 to 13.57
**Time in clinical practice is predominantly (> 50%) in palliative care**		
No	Ref	–
Yes	5.54	0.81 to 10.28
**Hours worked per week**		
≤ 40	Ref	–
41 to 60	3.08	−1.24 to 7.41
> 60	8.26	1.96 to 14.57
**Spends ≥ 20% of work time on non-clinical activities**		
Yes	Ref	–
No	−3.02	−6.94 to 0.90

There was a strong association between manifestation of burnout and resilience scores, with those meeting high burnout score criteria reporting significantly lower levels of resilience (Mean CD-RISC = 67.5, SD 11.8) compared to those who did not meet high burnout score criteria (Mean = 77.4, SD 11.2), *p* < 0.0001.

Twenty-five (15.15%) participants included additional comments at the end of the survey. Comments ranged from a few words, to a few sentences, to paragraphs and longer narratives. Comments were carefully reviewed and coded into initial codes and then thematic codes [44–47]. Thematic codes offered important insights on: the unique “double-edged sword” context of palliative care provision- meaningful, sustaining and impactful work with patients while also needing to advocate and fight for administrative, organizational, and political support to be able to provide optimal treatments, services and supports. A number of participants noted their exhaustion in having to continually push against myths and reluctance that other care givers/providers have about the value and importance of palliative care. Lastly, some participants shared their own personal and professional experiences with burnout, the support that was missing, and the lessons they had learned in building resilience and taking care of themselves. Comments about the concepts, instruments and application to palliative care were also carefully reviewed (Table 7).

**Table 7:** Thematic Coding of Open Text Comments (*N* = 25)

Themes	Quotes
**Unique context of palliative care** – Meaningful, sustaining and impactful work with patients while also needing to advocate and fight for administrative, organizational, and political support to be able to provide optimal treatments, services and supports	“I find working in Palliative Care a double-edged sword. Positive: the most fulfilling, impactful work that I have ever done. Negative and Irony: The personal costs of this work is massive. Contributors: Having no control over treatment/admissions decisions, Timeline and Acuity of Patients, Current system and supports for patients (lacking), Stress/Burnout of Self/Colleagues, Empathic suffering. My connection, empathy, and caring with patients and families is what makes me great at my job, but unable to sustain it. These patients can be very sick, and the timelines are short, so reversal/treatment is urgent. I feel tremendous emotional distress when I am unable to get what I perceive to be to be the most appropriate or timely procedure/intervention/action for my patient. The impact for the patient can be huge, and I feel powerless, emotionally distressed” (P-8) “I have read that the majority of burnout in Palliative Care Physicians comes not from the emotional toll our jobs take dealing with death and dying all the time but from the actual amount of work we have (increased number of complex patients, increased consult numbers, etc.) I find that is certainly true in my case and the cases of many of the physicians around me. Palliative Care definitely gives more immediate rewards and job satisfaction than (my other speciality), so I do not find the job itself increases burnout but the amount of work thrust upon me and the huge lack of resources and lack of Palliative Care Physicians in my province does contribute daily towards a feeling of burnout.” (P-14) “what provides me with the biggest “stress” is the constant fight for palliative care and its value and the constant push towards medical assistance in dying at the expense of palliative care. There is a need for some of us to advocate for the survival of palliative care but the climate at this point in time is hard to handle. There also is a lack of “recognition” within the medical profession for the work that palliative care physicians do.” (P-10) “The patients are uplifting, the administration not” (P-1) “The most difficult part of the job isn’t the patients – it’s the politics and administration” (P-21)
**Conflicts between caregivers and care providers** Conflicts as a result of other caregivers and care providers’ about the value and importance of palliative care	“personnellement, ce qui est. le plus difficile pour moi en soins palliatifs, et ce qui m’apporte le plus d’épuisement, est. la présence de conflits entre soignants face aux mythes et réticences encore présents envers les soins palliatifs” ^**a**^ (P-25) “I am often exhausted due to the poor opinion other areas have of Palliative Care especially “rich” areas such as Oncology and Cardiology where we get to deal with their patients at the most difficult time … It is rarely the work or patients that wear me out. It is the bureaucratic fights such as double rooms, non-functioning air conditioners, no view for the patients, terrible mattresses etc. that I find very hard to deal with.” (P-2) “Advocacy is one of my main roles, and it is a constant dance - trying to convince other physician subspecialty groups (with the power to make these decisions) to engage, act, treat …. during the day I will have threatened my physician-physician relationships by strongly advocating for my patients.” (P-8)
**Experiences of stress, fatigue, burnout** Present & Past Experiences of burnout	“I feel that PallCare is so protected compared to my previous speciality. It is hard to ever feel stress or burnout, as I know what that feels like in my Medicine world, rarely sleeping with 10–15 admissions a day.” (P-6) “I feel extremely burned out from my Palliative Care work. I am very close to quitting Palliative Care entirely.” (P-8) “Thanks for doing this. I am currently off work on stress leave due to professional burnout. Hoping I will be able to return but some things will need to change in the work environment.” (P-20) “Very glad to participate in this survey as this has been a very real concern for me in my personal practice lately. I look forward to hearing the results of the survey.” (P-19) “I want to comment that as a young physician working less than < 5 years total, my answers would have been drastically different one year ago when I burned out a couple of times in the first few years of practice. We do not learn how to practice resiliency and avoid burnout in medicine, let alone palliative care. We need to make this a focus for our new trainees as well as the current practicing physicians, who need to guide the trainees. I do not feel that I received any training on resiliency or burnout nor did I receive my institutional support when I burned out. I hope your study shows that even though burnout/resilience may be at the same rate as other physicians, as pall care physicians, we need to be able to support our colleagues.” (P-7) “You didn’t ask if I had experienced burnout in the PAST-to my surprise, I did and learned a number of my palliative colleagues had as well.” (P-17) “I did experience burnout in medical school, therefore learned to be resilient as I’ve progressed in my medical training and practice.” (P-3)
**Resilience-building – supporting self, having very firm personal and professional boundaries**	“This is very beautiful but also very brutal work. If we do not take the time to look after ourselves, in a real and meaningful way, this work will consume us … take the time to enjoy the happiness, love and sorrows in life is integral to living with purpose.” (P-5) “I have found it necessary to have very firm personal and professional boundaries in order to protect my physical and mental health. I still feel stressed a few times per month by having to defend those boundaries against judgments and pressures from colleagues. It would be very helpful if there were enough staff to do the amount of work to which our palliative program has committed. As it is, the demands are often unreasonable, and the choice facing the physician is to say no and feel that they are judged as uncooperative or lazy; or to say yes and feel the health effects of working excessively. That atmosphere is very jading.” (P-11) “Ma perception de stress et de fatigue évolue au fil d’une semaine de travail en soins palliatifs. Ça fait du bien d’avoir des congés, des pauses, afin de pouvoir être efficace, aidante et concentrée sur la tâche … Ma vie a totalement changé et je me sens nettement mieux; je n’aurais pas du tout répondu de la même façon à ce questionnaire il y a un an, étant sur le point d’un burn-out à ce moment.” ^b^ (P-24) “… I perceive that my past failures (rather than my past successes) have provided me with more resilience, specifically, overcoming past failures.” (P-3)
**Comments on survey in use of concepts, language and use of assessment tools**	“There are ways in which these inventories may fail to (fully) capture the experience of palliative care physicians … Frequent exposure to dying patients in an education in how little control we have in our lives, but I don’t feel particularly distressed by this.” (P-4) “The terms and concepts used fail to accept other belief systems, and use concepts that are foreign to my understanding of eastern spirituality and religion, potentially biasing your outcomes.” (P-9)

*P* Palliative Care Physician Participant

^**a**^ ***English Translation*** - Personally, what is most difficult for me in palliative care, and what brings me the most exhaustion, is the presence of conflicts between caregivers resulting from their misconceptions and hesitancy about palliative care

^b^ ***English Translation*** -My perception of stress and fatigue changes over the course of a week of work in palliative care. It feels good to have time off, breaks, to be able to be efficient, helpful and focused on the task. (Since I reorganized my work schedule to alternate sites in palliative care, in addition to time for rest), my life has totally changed and I feel much better; I wouldn’t have answered this questionnaire at all the same way a year ago, being on the verge of a burnout at the time

## Discussion

In addressing our overarching study question about the prevalence of burnout and degree of resilience in Canadian palliative care physicians, the 38.2% burnout rate in this pan-Canadian study is comparable with similar palliative care physician studies in the United States using similar MBI-HSS (MP) cut-offs [28], and greater than reported in other countries, notably Australia [29] and France. Important to highlight is the variability that continues to exist around prevalence rates, how burnout measurement cut-off points are utilized and how total scores and subscale results are reported, and lastly, how burnout is defined [25, 27]. Comparing MBI-HSS (MP) subscale results, a substantial proportion of respondents (36.4%) scored high on emotional exhaustion (EE), compared to much lower proportions who scored high on depersonalization (DP) (15.1%) or low on personal accomplishment (PA) (7.9%). In fact, most respondents (64.2%) scored low on depersonalization and high on personal accomplishment (72.1%), suggesting that most feel connected and engaged with their patients, and are fulfilled in their work, consistent with their professional career choice. However, many respondents feel emotionally exhausted, which may be due to high workloads and demands, and may also involve cumulative emotional burden and grief.

While palliative care physicians may have protective factors that are speciality specific, which speak to advanced training around communication, dying and death and grief [54, 55], our study findings highlight that further research is required to understand what factors heighten and compound experiences of emotional exhaustion and low levels of resilience. Further illuminated in our open-text review, specific participants commented on the meaningful and sustaining work of delivering palliative care while also directly acknowledging the personal costs, accumulated stressors in delivering care with limited time, resources and support, and the persistent conflicts in advocating for the legitimacy and importance of providing palliative care. These findings, echo the importance of examining individual (personal and coping strategies), interpersonal and organizational (systemic stressors) effects of burnout and protective factors that raised in other studies with hospice and palliative care clinicians [35, 50]. Further, as demonstrated in previous studies [41, 42], there was a strong association between burnout and resilience: respondents reporting lower levels of resilience had higher levels of burnout. While causality cannot be determined, this result suggests that resilience-building can be a valuable tool in burnout prevention, a component that is often included in resilience-building interventions with clinicians [56].

In this study, physicians age > 60 reported lower burnout levels and higher levels of resilience. This correlation is consistent with other studies examining physician burnout [29, 41, 57] and resilience [36]. The particularly low levels of burnout in those aged > 60 (3.4%), compared to other age groups in our study, under 40 (41.7%), 41–50 (48.6%), and 51–60 (47.1%), is suggestive of some form of survivorship occurring for those who are later in their career. Recognizing that older physicians experience lower levels of burnout and higher levels of resilience suggests that early to mid-career physicians may benefit from mentoring, coaching and peer-to-peer support programs with later-career palliative care physicians [58, 59]. Further study is required to fully understand this correlation as this may be reflective of accumulation of experience over time and development of a repertoire of resilience strengthening attitudes, coping strategies and behaviours to manage stressors. It may also be reflective of “survivor bias,” where those who have experienced significant burnout have left the field. Study trends which highlight factors and elements that require further study, such as the length of time in practice, number of hours worked, and focus of clinical practice, can help us to understand how these elements work together to mitigate or protect palliative care physicians from burnout. This understanding may offer important insights about not only what physicians are experiencing, but also how we can best support physicians as the move through their medical career. Although some studies have found a risk for women physicians to experience higher rates of burnout [28, 36] and lower levels or resilience [36], this was not seen in our study.

Burnout prevention will likely require increasing resilience skills on individual levels as well as interventions which modify workplace factors on an organizational level. Leiter and Maslach identified six areas of work environment relevant to how clinicians experience work demands: workload, control, reward, community, fairness, and values [60]. West et al. further identified that many of the drivers of the burnout epidemic are rooted within healthcare organizations and systems including excessive workloads, inefficient work processes, clerical burden, lack of input or control, suboptimal organizational support and leadership culture [61].

While recognizing that generic open-text comments in surveys need to be reviewed carefully, and acknowledging that there are limits around the depth and breadth of understanding that can be acquired through open-text comments [62, 63], these comments also provided important insights around how some palliative care physicians understand and make meaning of their experiences with burnout and resilience. Thematic coding offered the ability to further enhance our quantitative findings in beginning to explore how organizational, administrative, political and cross-disciplinary elements of palliative care provision may cause stress, exhaustion, distress and burnout. Further identified were personal and professional comments about present-day stress, fatigue, and burnout, in addition to past experiences of burnout and how some participants were building resilience into their lives. Many of these issues have been identified in other studies [50, 64, 65], and further research using mixed-methods approaches is needed to better understand what contributes and mitigates experiences of burnout and resilience and importantly, what system-wide changes and potential interventions are required, to best support physicians across specialities [56, 61, 66]. Most importantly, it is critical that research in this area is integrated into policy and practice, to ensure that palliative care physicians are supported to provide compassionate, competent, comprehensive care.

### Limitations

One limitation of this study is the response rate (29%). However, our response rate is higher than in comparison with other national surveys of burnout across all physician groups (17.1% in the United States [10] and 8.5% in Canada [36]). It is also possible that some palliative care physicians are non-members of either society and therefore would not have been invited to participate, introducing sampling bias. Furthermore, we did not have access to the demographics of either membership rosters for comparison with our study sample, further limiting the study’s generalizability. The cross-sectional study design precludes a determination of causality or direction of effect for the associations observed. We were also unable to capture variability in experienced burnout over time, including past experiences with burnout. Variations in MBI-HSS (MP) cut-off scores used to define burnout is another limitation in this area of research: our study results were only comparable to other studies with similar MBI-HSS (MP) cut-offs. A systematic review on the prevalence of burnout among physicians found that of studies using MBI-HSS (MP), there were at least 47 distinct definitions of burnout, highlighting the importance of developing a consensus definition of burnout [67]. We recognize the limitations that exist around how and in what way open text comments can be analysed, particularly when these were not framed a priori*,* specific questions were not asked directly, and open-text items in surveys have both explicit and implicit constraints in really understanding participants’ expereinces [62, 63]. Despite these cautions, participants who did provide open-text responses offered very important, rich and fulsome accounts of their perceptions and experiences, and we believe these reflection offer important insights which offer further support to our quantitative findings, while also offering direction for future research areas to explore.

## Conclusions

In our study, it is concerning that more than one third of Canadian palliative care physicians reported high levels of burnout and many of these physicians feel emotionally exhausted and overextended. Higher scores on personal accomplishment, and lower scores on depersonalization highlight that palliative care physicians may experience a number of protective and resilience-building factors, but these factors need to be contextualized within broader care delivery stressors, related to control over workload, time, resources, and appreciation and respect for the profession of palliative care. Further research is required in understanding how palliative care physicians who have been practicing longer in the field have learned to not only build but sustain resilience over time, and importantly what learning can be shared with early and mid-career physicians to further support resilience-building factors at individual, interpersonal and organizational levels. Clearly highlighted is the importance of further study in learning about how palliative care physicians experience care delivery, the meaning and importance of this work in their lives overall, and how important it is that this learning is translated into policy and practice contexts.

## Supplementary Information

**Additional file 1: Supplementary Figure S1. CSPCP:** Canadian Society of Palliative Care Physicians; **SQMDSP:** Société Québécoise des Médecins de Soins Palliatif.

**Additional file 2: **Multivariable linear regression, estimating difference in mean Connor-Davidson Resilience Scale (CD-RISC) total score (*n* = 165) [with years of practice and not age in the model].

## Data Availability

The data that support the findings of this study are available on reasonable request from the corresponding author [ES]. The data are not publicly available due to them containing information that could compromise research participant privacy/consent.

## Abbreviations

MBI-HSS (MP): Maslach Burnout Inventory for Medical Professionals
CD-RISC: Connor-Davidson Resilience Scale
EE: Emotional Exhaustion
DP: Depersonalization
PA: Personal Accomplishment
CSPCP: Canadian Society of Palliative Care Physicians
SQMDSP: Société Québécoise des Médecins de Soins Palliafs
CHERRIES: Checklist for Reporting Results of Internet E-Surveys
OR: Odds Ratio
CI: Confidence Interval
