# Supplementary material for: Burnout and resilience among Canadian palliative care physicians
Source: BMC Palliat Care. 2020 Nov 6;19:169. doi: 10.1186/s12904-020-00677-z (PMC7648393; doi:10.1186/s12904-020-00677-z)
Supplement: Supplementary file 1 — Additional file 1: Supplementary Figure S1. CSPCP: Canadian Society of Palliative Care Physicians; SQMDSP: Société Québécoise des Médecins de Soins Palliatif. [file 12904_2020_677_MOESM1_ESM.docx]

**Supplementary Figure S1**

CSPCP members (N=622)

CSPCP members invited (N=479)

- Practicing physicians (N=415)
- Residents (N=63)
- Retiree invited by mistake (N=1)

Non-physician, retired, or physician outside of Canada (N=45)

Not willing to participate in surveys (N=99)

Non-physician, retired, or physician outside of Canada

(N=3)

SQMDSP members (N=93)

SQMDSP members invited (N=90)

- Practicing physicians (N=86)
- Residents (N=4)

**CSPCP:** Canadian Society of Palliative Care Physicians; **SQMDSP:** Société Québécoise des Médecins de Soins Palliatif.
