# Supplementary material for: Burnout and resilience among Canadian palliative care physicians
Source: BMC Palliat Care. 2020 Nov 6;19:169. doi: 10.1186/s12904-020-00677-z (PMC7648393; doi:10.1186/s12904-020-00677-z)
Supplement: Supplementary file 2 — Additional file 2: Multivariable linear regression, estimating difference in mean Connor-Davidson Resilience Scale (CD-RISC) total score (n = 165) [with years of practice and not age in the model]. [file 12904_2020_677_MOESM2_ESM.docx]

**Additional File 2: Multivariable linear regression, estimating difference in mean Connor-Davidson Resilience Scale (CD-RISC) total score (n=165) [with years of practice and not age in the model]**

|  | **Mean difference** | **95% CI** |
| --- | --- | --- |
| **Gender**  Male  Female | Ref  0.28 | -  -4.12 to 4.70 |
| **Years in practice**  ≤10  11 to 20  21 to 30  >30 | Ref  -2.98  -0.01  4.59 | -  -8.17 to 2.21  -5.00 to 5.01  -0.96 to 10.14 |
| **Time in clinical practice is predominantly in palliative care**  No  Yes | Ref  5.18 | -  -0.37 to 9.98 |
| **Hours worked per week**  ≤40  41 to 60  >60 | Ref  2.23  7.03 | -  -2.11 to 6.57  0.67 to 13.40 |
| **Spends ≥20% of work time on non-clinical** **activities**  No  Yes | Ref  -2.48 | -  -6.46 to 1.50 |
